# Transcriptomic response in planktonic and biofilm-associated cells of *Streptococcus mutans* treated with sublethal concentrations of chlorhexidine

**DOI:** 10.1093/femsle/fnaf100

**Published:** 2025-09-23

**Authors:** Sara Arbulu, Thomas F Oftedal, Morten Kjos

**Affiliations:** Faculty of Chemistry, Biotechnology and Food Science, Norwegian University of Life Sciences, 1432 Ås, Norway; Faculty of Chemistry, Biotechnology and Food Science, Norwegian University of Life Sciences, 1432 Ås, Norway; Faculty of Chemistry, Biotechnology and Food Science, Norwegian University of Life Sciences, 1432 Ås, Norway

**Keywords:** oral biofilms, *Streptococcus mutans*, RNA-seq compendium, transcriptome, sublethal antibiotics, chlorhexidine, amoxicillin

## Abstract

Chlorhexidine, an antimicrobial with a broad inhibitory spectrum, is commonly used to treat oral infections as an active ingredient in mouthwash. While typically used at high concentrations (1–2 mg/ml), oral bacteria can be exposed to sublethal concentrations due to the bioavailability and protective barrier of biofilms (dental plaques). Sublethal concentrations can cause transcriptional remodelling of bacteria such as *Streptococcus mutans*, a key player in dental caries. Using an RNA-seq approach, this report provides a compendium on the effect of sublethal concentrations of chlorhexidine on the transcriptome of *S. mutans* as planktonic cells and in biofilm states. *Streptococcus mutans* showed major transcriptional remodelling between planktonic and biofilm states. The transcriptional response towards chlorhexidine was more pronounced in planktonic cells compared to sessile cells. However, the response observed for biofilm-associated cells was not specific to chlorhexidine, as the transcriptional response in biofilms exposed to the β-lactam amoxicillin was similar to those observed for chlorhexidine. Furthermore, we found that *S. mutans* modulates the transcription of a multitude of ABC transporters in both planktonic and biofilm-associated cells upon exposure to these antimicrobials.

## Teaser

Streptococcus mutans, an oral microbe related to dental caries, showed significant gene expression changes in both free-floating and biofilm states when exposed to a sublethal concentration of chlorhexidine.

## Introduction

Dental caries is the gradual loss and demineralization of the hard tissues of teeth caused by acid production from biofilm-associated bacteria in the oral cavity (Pitts et al. 2017). If untreated, caries can lead to severe infection and require antibiotic treatment. Dental caries can be prevented by frequent disruption of biofilms in the oral cavity by brushing and flossing and by the use of antiseptic mouthwashes. Clinically relevant concentrations of these antimicrobials are likely to lead to sublethal concentrations at certain target sites due to decreased bioavailability and accessibility (Delacher et al. 2000, Liu et al. 2024). This is particularly relevant for biofilm-related conditions such as dental caries, the most prevalent microbe-related health condition worldwide (Ward and Goldie 2024).

Chlorhexidine and amoxicillin are commonly used to control oral infections with different clinical indications (Brookes et al. 2020, Abdullah et al. 2024). Chlorhexidine is often used as a mouthwash (containing 0.1%–0.2% chlorhexidine digluconate) by dental clinicians during presurgical preparation to reduce the bacterial load and after surgery as a preventative measure (Brookes et al. 2020, Poppolo Deus and Ouanounou 2022). Furthermore, in many regions, chlorhexidine is available as an over-the-counter mouthwash and used to manage early gum disease (gingivitis) or used as a prophylactic and adjunct to brushing to prevent plaque formation (Brookes et al. 2020). Chlorhexidine is a cationic agent that interacts with the negatively charged bacterial membranes, altering their osmoregulation and leading to leakage of ions and cellular components that eventually leads to cell lysis (Lim and Kam 2008). Chlorhexidine is a broad-spectrum antiseptic that is active against bacteria, viruses, and fungi and can also disrupt biofilms (Bonez et al. 2013, Karpiński and Szkaradkiewicz 2015, Alvendal et al. 2020, Poppolo Deus and Ouanounou 2022). While bacteria typically are exposed to chlorhexidine for a few minutes during mouthwashes, chlorhexidine shows substantivity, meaning that it binds to the oral tissues after administration and is slowly released over time. This maintains the antimicrobial activity for hours after rinsing with concentrations gradually decreasing to subinhibitory levels (Brambilla et al. 2004, Cousido et al. 2010, Reda et al. 2020). On the other hand, orally administered amoxicillin is the most common choice for systemic tooth infection treatment (Akhavan et al. 2024). Amoxicillin is a β-lactam that targets peptidoglycan synthesis in actively dividing cells, and it displays activity against Gram-positive bacteria, including *Streptococcus*, and some Gram-negative bacteria such as *Escherichia coli* (Castle 2007).

Sublethal concentrations of antibacterials affect the physiology of the cells and can select for resistant bacteria, promote genetic variability, and function as signalling molecules affecting virulence, biofilm formation, and communication mechanisms (Andersson and Hughes 2014, Silva et al. 2014, Waack and Nicholson 2018, Liu et al. 2020, Penesyan et al. 2020, Guo et al. 2021, Byun et al. 2022). Understanding those effects as part of the antibiotic response can help design optimized therapies and gain an increased understanding of the ecology of these bacteria.

While most research on the effect of antimicrobials has been conducted on planktonic cells, pathogens such as the oral bacterium *Streptococcus mutans* often form biofilms during or as part of their infection cycle. *S. mutans* is key to dental caries development and oral biofilm formation (Lemos et al. 2019). By metabolizing carbohydrates in the oral cavity, *S. mutans* produces glucans that contribute to forming the biofilm extracellular matrix. As a by-product, organic acids that lower the pH are produced, leading to tooth decay. However, this process is only part of the explanation for the complex, multifactorial, and multispecies aetiology of dental caries (Cai and Kim 2023).

Biofilm-associated cells differ from planktonic cells in their response to antibiotics (Shree et al. 2023) and generally display greater heterogeneity (Obando and Serra 2024). Bacteria embedded in biofilm matrices are more resilient to antibiotic treatment due to limited diffusion of antibiotics and slower growth rates of the cells (Cozens et al. 1986, Lebeaux et al. 2014). Consequently, antibiotic concentrations effective against planktonic cells become subinhibitory for biofilm-associated cells in dental caries.

Studies examining global gene expression of *S. mutans* under clinical conditions are limited. Previous RNA-seq studies have examined subinhibitory effects of chlorhexidine on planktonic *S. mutans* (Muehler et al. 2022), the transcriptomes of naturally occurring biofilms (He et al. 2017, Santos et al. 2022) or focused on the expression of specific genes (Shemesh et al. 2007), while others have relied on phenotypic methods to assess the antimicrobial effects of oral medications (Clark et al. 2017). In this work, we challenged *S. mutans* UA159 planktonic and biofilm-associated cells with a sublethal concentration of chlorhexidine for a short duration to mimic exposure from the use of mouthwash. We then used RNA-seq to analyse and compare differential gene expression changes. Amoxicillin was also applied to the biofilm cultures for comparison purposes. The results showed an overall differential regulation of planktonic and biofilms with extensive downregulation of chlorhexidine and amoxicillin-treated biofilm metabolism.

## Materials and methods

### Bacterial strains and antibiotics used

*Streptococcus mutans* UA159 was cultured in Brain Heart Infusion (BHI, Difco) for planktonic growth in broth and in BHI supplemented with 1% sucrose (BHIS) for the biofilm experiments. The strain was grown at 37°C in anaerobiosis (10% CO_2_) or airtight tubes, unless otherwise stated.

Chlorhexidine digluconate 20% (Sigma) was prepared and diluted to the desired concentration in sterile Milli-Q water. For amoxicillin, stocks at 10 mg/ml were prepared by dissolving amoxicillin trihydrate in an equal volume of 0.1-M NaOH and phosphate-buffered saline (pH 7.2) and further diluted in Milli-Q water to the desired concentration.

### Antibiotic susceptibility tests to amoxicillin and chlorhexidine

#### Minimum Inhibitory Concentration determination

The experiments were set up in 96-well polystyrene microtiter plates (Sarstedt, catalogue number 821 581 001) with a total volume of 300 µl using the broth microdilution method. Two-fold dilution series of chlorhexidine (starting concentration 100 µg/ml) and amoxicillin (starting concentration 100 ng/ml) were prepared in BHI, with each well containing 150 µl of the diluted solutions. Subsequently, 150 µl of *S. mutans* UA159 culture at an OD_600_ of 0.05 was added to each well. The cultures were incubated at 37°C, and the plate was shaken for 5 s before measurements of OD_600_ were taken every 10 min throughout the experiment using a Hidex Sense (Hidex Oy) plate reader. The experiments were repeated three times. Minimum Inhibitory Concentration (MIC) was established as the lowest concentration of antimicrobial that inhibited bacterial growth.

#### Tolerance to chlorhexidine during growth

*Streptococcus mutans* UA159 culture at OD_600_ 0.05 was incubated at 37°C until it reached an OD_600_ of 0.3–0.4. At this point, chlorhexidine was added. The same two-fold dilution series used for the MIC assays was tested (100, 50, 25, 12.5, 6.25, 3.125, 1.5, 0.78, 0 µg/ml). Growth was monitored in a plate reader as described above.

#### Biofilm-Oriented-Antimicrobial Test

The metabolic activity of *S. mutans* UA159 biofilm-cells treated with chlorhexidine and amoxicillin was determined by the Biofilm-Oriented Antimicrobial Test (BOAT) (Grønseth et al. 2017, Kranjec et al. 2020). *Streptococcus mutans* UA159 culture at an OD_600_ of 0.5 was diluted 1:1000 in BHIS, and 100 µl was added to the wells of a 96-well plate and allowed to form biofilms for 24 h at 37°C in anaerobiosis. Different concentrations of chlorhexidine (800, 775, 750, 725, 700, 650, 600, 120, 100, 71, 50, 42, 24, 14, 8, 6, 5, 3, 2 µg/ml) and amoxicillin (5000, 2500, 1250, 625, 313, 156, 78, 39, 20, 10, 5, 2, 1 µg/ml) were prepared in BHI to a volume of 175 µl. These concentrations were selected after several rounds of BOAT assays to show the effect of a wide range of antibiotic concentrations on *S. mutans* UA159 biofilms. Biofilms were washed twice with 100 µl of 0.9% NaCl and 150 µl of the antibiotic dilutions were transferred to the biofilm plate. Antibiotic treatment was applied for 5 min, 30 min, and 24 h while incubating at 37°C in anaerobiosis. The biofilms were washed three times with 0.9% NaCl and 100 µl of 0.025% triphenyl-tetrazolium chloride (TTC, Sigma) dissolved in BHI was added to each well and further incubated at 37°C for 5 h. The presence of red colour was used as a measure of cellular respiration. TTC was removed, and 200 µl ethanol:acetone (70:30) were added per well and incubated overnight to extract the red dye. The metabolic activity was then measured at 492 nm in a plate reader (Fluostar Optima, BMG, LabTech). Biofilms without antibiotic treatment were used as controls. Three replicates per condition were performed.

### RNA isolation and sequencing

RNA was isolated from planktonic broth and biofilm cultures of *S. mutans* UA159 challenged with sublethal concentrations of amoxicillin and chlorhexidine for 5 min at 37°C in anaerobiosis. In broth, chlorhexidine was used at a final concentration of 6.25 ng/ml. In biofilm experiments, chlorhexidine and amoxicillin were used at 70 µg/ml and 5 mg/ml, respectively. Concentrations used were based on MIC and BOAT assays.

The initial broth culture of *S. mutans* UA159 was prepared by inoculating 50-ml BHI with 0.5 ml of a starting inoculum with an OD_600_ of 0.4–0.5. Growth was monitored until an OD_600_ of 0.3 (early-mid exponential phase). Aliquots of 10 ml were used for chlorhexidine treatment and as a control (treated with BHI). The cells were harvested by centrifugation at 6000 x g for 1 min at 4°C. The pelleted cells were immediately frozen in liquid nitrogen and stored at −80°C. This experiment was repeated three times (biological replicates).

For the biofilm cultures of *S. mutans* UA159, 20 µl of a starting culture with an OD_600_ of 0.4–0.5 was inoculated in 20-ml BHIS. The biofilm was grown in 48-well polystyrene plates (Nunc, catalogue number: 140 675) with 400 µl of culture per well and allowed to grow for 24 h at 37°C in anaerobiosis. Planktonic cells were removed by aspiration and attached cells/biofilms were washed once with 400 µl 0.9% NaCl and then 400 µl of amoxicillin or chlorhexidine at the concentrations mentioned above. The control with no antibiotic was treated with BHIS. Four wells per condition were used. After the antibiotic treatment, the biofilms were washed with 400-µl sterile RNAse-free water (Invitrogen). Then 400 µl of RNAprotect (Qiagen) was added to the wells, and the biofilms were scraped off the surface using a pipette tip (Kragh et al. 2019). The suspended biofilms from the four wells for each condition was transferred to 15-ml tubes and centrifuged at 6000 x g for 1 min at 4°C. The pelleted cells were immediately frozen in liquid nitrogen and stored at −80°C. The experiment was repeated three times (biological replicates).

RNA was extracted using the RNeasy Mini Kit (Qiagen), followed by DNase treatment and phenol-chloroform extraction, as described by Stamsås et al. (Stamsås et al. 2018). Library preparation, quality assessment, and sequencing were conducted by Novogene (Germany).

In summary, rRNA was removed from the total RNA using the Illumina Ribo-Zero Plus rRNA Depletion Kit, followed by ethanol precipitation. Second-strand cDNA synthesis incorporated dUTPs instead of dTTPs to generate a directional (stranded) library. The sequencing library was made using Novogene’s NGS Stranded RNA Library Prep Set (PT044). Library quantification was performed using Qubit and real-time PCR, while size distribution was assessed with a Bioanalyzer. The quantified libraries were pooled and sequenced on a Novaseq 6000 Illumina instrument. The raw sequencing reads were processed to remove adapters, reads with > 10% ambiguous bases (N), and low-quality reads (Qscore ≤ 5).

The reads were then aligned against *S. mutans* UA159 (Genbank accession number: AE014133.2), and differentially expressed genes (DEGs) between planktonic, biofilm, treated, and untreated samples were calculated using DESeq2 (Love et al. 2014) on three independent biological replicates for each tested condition using the Bioconductor R package. See Table 1 for the conditions being analysed and compared. An additional rRNA removal step was done bioinformatically by filtering out ribosomal locus tags based on the reference genome. Genes with an adjusted *P*-value ≤ 0.05 were used for further analysis. DEGs were scored as upregulated if they had a log_2_fold change ≥ 1.0 and downregulated if they had a log_2_fold change ≤ −1.0. Visualizations of DEGs were done as volcano plots and a heatmap using the EnhancedVolcano and heatmap R packages, respectively. A Venn diagram was also constructed to identify the common DEGs obtained from the different compared conditions using the gvenn R package.

**Table 1.** Analysed and compared conditions for RNA-seq analysis of *S. mutans* UA159 planktonic and biofilm cultures.

Analysed conditions^a^	
*Planktonic broth cultures*	*Biofilm cultures*
–Untreated	–Untreated
–CHX treated	–CHX treated
	–AMOX^b^ treated
**Compared conditions**	
*Culture condition 1*	*Culture condition 2*
Untreated planktonic vs	Untreated biofilm
Untreated planktonic vs	CHX-treated planktonic
Untreated biofilm vs	CHX-treated biofilm
Untreated biofilm vs	AMOX-treated biofilm
AMOX-treated wt biofilm vs	CHX-treated biofilm

a CHX, chlorhexidine; ^b^AMOX, amoxicillin.

Transcripts per million (TPM) values were calculated based on the normalized counts provided by DESeq2. Bray–Curtis dissimilarity was used to compute distance matrices based on TPM values, and principal coordinates analysis (PCoA) was performed to visualize sample clustering. Additionally, a heatmap was calculated using the *pheatmap* R package to visualize expression patterns across samples, and a correlation matrix showed associations between samples using the *corrplot* R package.

### Functional annotations and pathway analysis

To obtain insights into the biological meaning of the DEGs clusterProfiler R package v4.13.0 (Yu et al. 2012) was used for gene set enrichment analysis using the Kyoto Encyclopedia of Genes and Genomes database via the functions *enrichKEGG* and *compareCluster* setting a *P*-value cut-off <0.05. Note that the global maps and the overview maps are a special class of metabolic pathway maps within KEGG (Kanehisa et al. 2017). These categories represent an integrated picture of the metabolism connecting different pathways present in the dataset.

### Sequencing data availability

The raw FASTQ data are accessible at https://www.ebi.ac.uk/ena/browser/home with accession number PRJEB83273.

## Results and discussion

### Selection of sublethal chlorhexidine and amoxicillin concentrations applied on planktonic and biofilm cultures of *S. mutans* UA159

**Figure 1.**
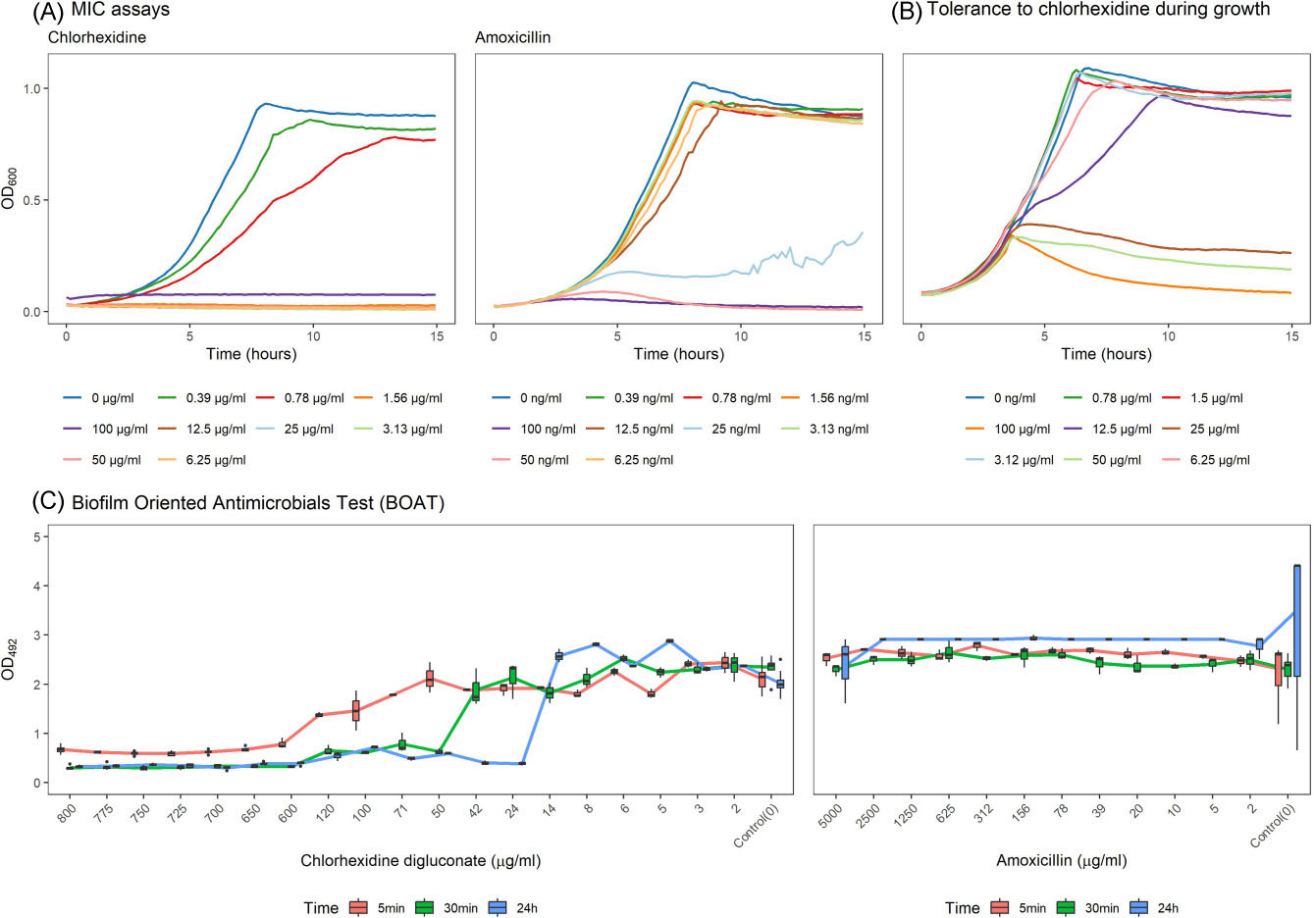
Determination of the sensitivity of planktonic and biofilm cultures of *S. mutans* UA159 to amoxicillin and chlorhexidine. (A) Minimal Inhibitory Concentration assays. (B) Tolerance to growth with chlorhexidine and amoxicillin. (C) Biofilm-Oriented Antiseptic Test assays. The boxplots show the remaining metabolic activity after antibiotic treatment at different concentrations at treatment durations (5 min, 30 min, 24 h). The median distribution is shown as a cross of the boxplots, and the degree of variability (interquartile region) is represented as the height of the boxes. The whiskers indicate the maximum and minimum values observed.

To select a relevant concentration of chlorhexidine that could exert changes at the transcriptome level, we first established the sensitivity of planktonic *S. mutans* UA159 to chlorhexidine by MIC assays (Fig. 1A and B). Complete inhibition of growth was observed at 1.56 µg/ml (Fig. 1A). This is in line with other studies, which have found the MIC of *S. mutans* for chlorhexidine to be below 1 µg/ml (Järvinen et al. 1993, Mohammed Ghilan et al. 2023). The same concentration range of 0.3–100 µg/ml was used to assay if *S. mutans* UA159 grown to early-mid exponential phase could tolerate the presence of chlorhexidine (Fig. 1B). At this phase of growth, *S. mutans* UA159 was only affected by chlorhexidine concentrations above 6.25 µg/ml. A concentration of 6.25 µg/ml was therefore used for the chlorhexidine transcriptomic stress response analysis in broth. The MIC for amoxicillin was determined to be 50 ng/ml, which is in line with other studies (Fig. 1A) (Kwon and Lee 2020, Maisonneuve et al. 2020).

We then examined the effect of chlorhexidine on *S. mutans* UA159 biofilms (Fig. 1C). Chlorhexidine is reported to disrupt *S. mutans* biofilms in a dose-dependent manner (Ccahuana-Vásquez and Cury 2010, Silva et al. 2014, Lee et al. 2016) favouring *S. mutans* biofilm detachment (Liu et al. 2012). To determine the concentration needed to disrupt *S. mutans* biofilms, we used the BOAT assay (Kranjec et al. 2020). Using this assay, we were able to quantify the remaining metabolic activity of 24 h-old biofilms treated with chlorhexidine for 5 min, 30 min, and 24 h (Fig. 1C). The metabolic activity was reduced according to concentration and treatment duration. For the 5 min treatment, a chlorhexidine concentration of 600 µg/ml was needed to fully reduce the metabolic activity, indicating a complete disruption of the formed biofilm. The corresponding concentrations for the 30 min and 24 h treatments were 50 and 24 µg/ml chlorhexidine, respectively (Fig. 1C). The highest concentration of chlorhexidine that did not appreciably affect metabolic activity after a 5 min treatment was determined to be 70 µg/ml, which we chose as the sublethal concentration for the transcriptomic experiments. This concentration falls within the range expected in the oral cavity after chlorhexidine mouthwash rinsing (Brambilla et al. 2004, Reda et al. 2020). We also attempted to determine a corresponding concentration for amoxicillin against biofilms; however, no effect was observed on the metabolic activity even with the highest concentration tested (5 mg/ml) (Fig. 1C). This is most probably due to the nature of the cells in the biofilms and highlights the difference in physiology between cells in biofilms compared to planktonic growth. Amoxicillin and other β-lactams inhibit bacterial cell-wall synthesis due to binding to the penicillin-binding proteins, thereby inhibiting transpeptidation of peptidoglycan. However, most cells in a biofilm are not actively dividing and relatively mature *S. mutans* biofilms allowed to establish for at least 24 h contain mostly inactive nondividing cells (Lewis 2005).

### Overview of *S. mutans* RNA-seq analysis

To investigate the transcriptomic response of planktonic and biofilm cultures of *S. mutans* UA159 upon 5-min treatment with sub-inhibitory concentrations of chlorhexidine and amoxicillin, an RNA-seq analysis was conducted. A 5-min treatment duration was selected to mimic clinical scenarios of residual chlorhexidine between rinsing, several hours postrinsing or microbial adaptation in biofilms where drug diffusion is limited.”

A total of 247 746 462 raw reads were generated, of which 98.10% were clean reads, with an average GC content of 39.96%, Q > 30 93.77%, and an underlying error of 0.03%, indicating the high quality of the sequencing data (Table S1). TPM values were used to compare the proportion of reads mapped to a gene in each sample (Table S2). PCoA of the gene data showed grouping of the biological replicates, with a clear separation between planktonic and biofilm samples and further separation between nontreated and amoxicillin and chlorhexidine-treated samples (Fig. S1). A heatmap of all genes and a correlation plot further confirmed the expected clustering of the samples (Fig. S2). No clear clustering was seen in the PCoA plot (Fig. S1), but the heatmap of differentially expressed genes (Fig. S2) showed clearer separation between chlorhexidine-treated and control groups, suggesting that chlorhexidine-induced changes are better detected by clustering methods that emphasize differential or correlated gene expression.

**Figure 2.**
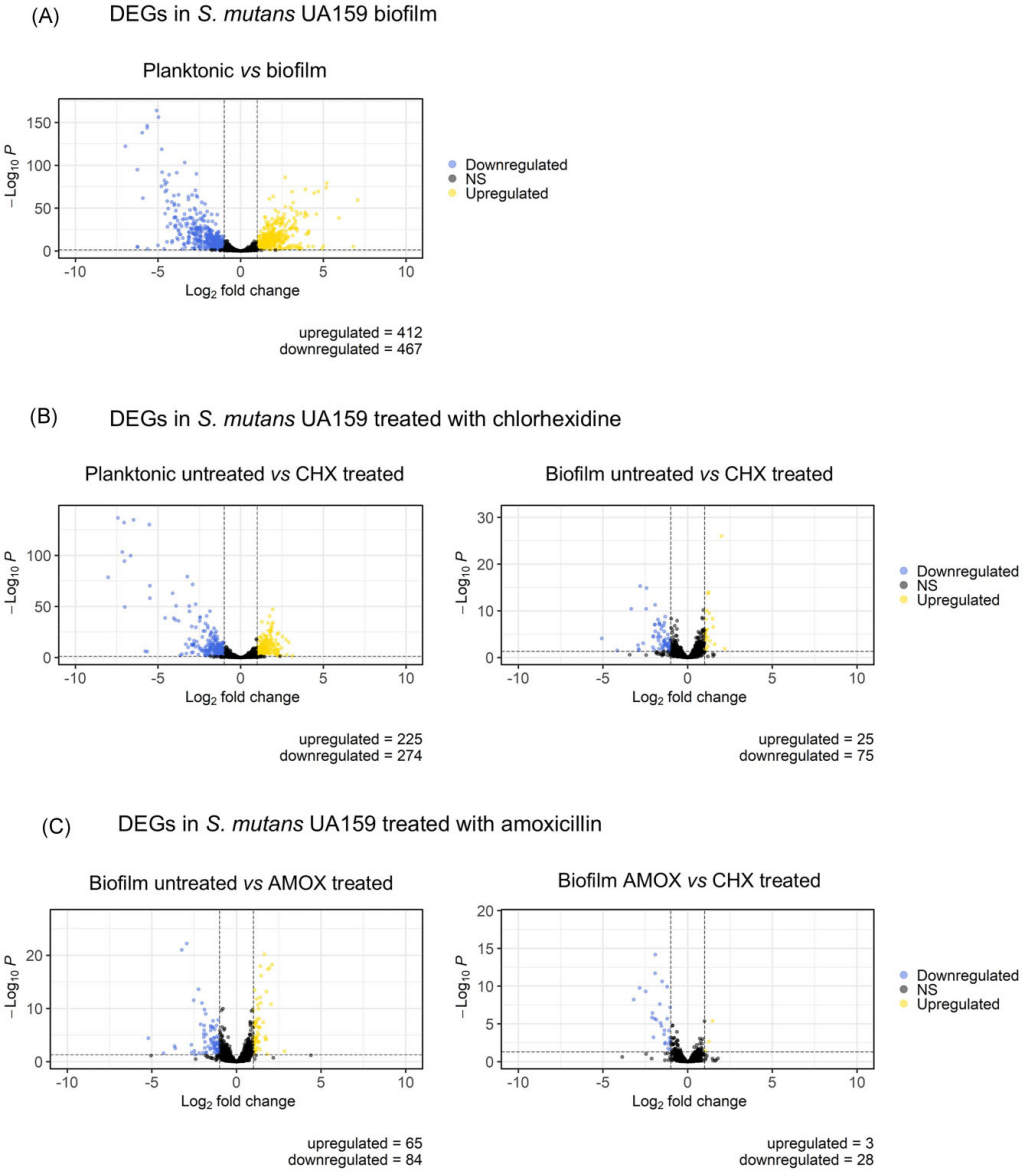
Volcano plots showing differentially expressed genes between planktonic and biofilm cultures of *S. mutans* UA159 treated with sublethal concentrations of amoxicillin and chlorhexidine. The *x*-axis represents the fold-change in gene expression between different conditions, and the *y*-axis represents the statistical significance of the found differences. Significantly up and down-regulated genes are filtered (|log_2_FC| > 1, P_adj_ < 0.05) and highlighted in yellow and blue dots, respectively. Genes that are not differentially expressed are shown in black. Abbreviations: CHX, chlorhexidine, AMOX, amoxicillin.

The distribution of differentially expressed genes (DEGs; |log_2_FC| > 1, P_adj_ < 0.05) between the compared conditions (Table 1) was visualized using volcano plots (Fig. 2). A list of all DEGs can be found in the supplementary material (Table S3).

### Biofilm transcriptional behaviour differs from planktonic cells of *S. mutans* UA159

**Figure 3.**
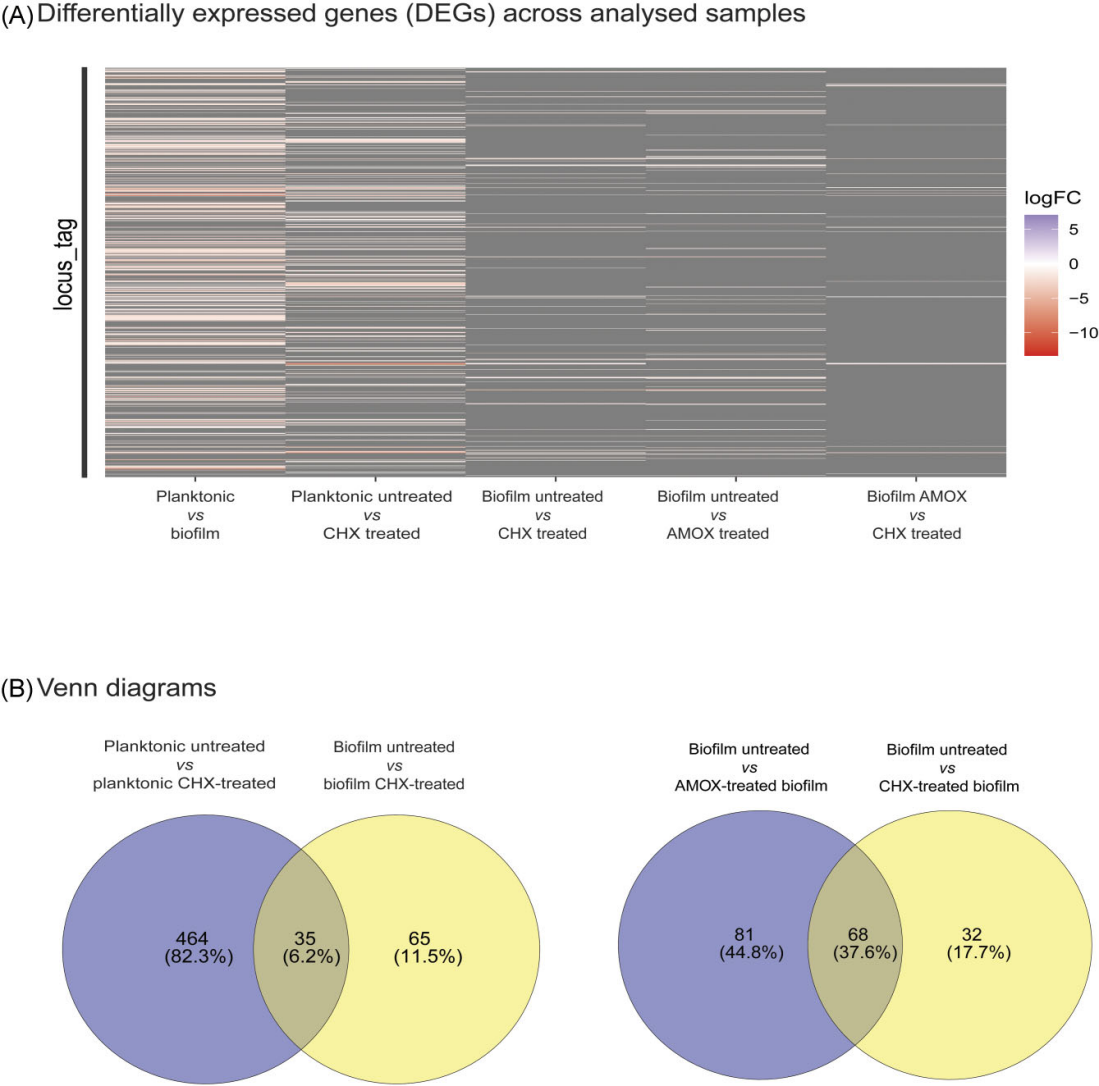
DEGs representations across compared conditions. (A) Heatmap of DEGs. (B) Venn diagrams comparing different conditions. Abbreviations: CHX, chlorhexidine, AMOX, amoxicillin

Gene regulation of planktonic and biofilm cultures can differ significantly reflecting the two different microbial lifestyles (Charlebois et al. 2016a, Sánchez et al. 2019a, Shemesh et al. 2007, Lo et al. 2009, Castro et al. 2017, Zheng et al. 2022). Indeed, with a threshold of |log_2_FC| > 1 and P_adj_ < 0.05, a total of 879 genes (412 upregulated, 467 downregulated genes, 43% of genes in total) were found to be differentially expressed in biofilm relative to planktonic cultures of *S. mutans* UA159 (Figs. 2A and 3A). Previous DNA-microarray analyses showed about 12% of *S. mutans* UA159 genes to be differentially expressed in biofilms (Shemesh et al. 2007), whereas studies in other species showed broader variability, from 1% differential expression in *Pseudomonas aeruginosa* (Whiteley et al. 2001), 4.8% in *Porphyromonas gingivalis* (Sánchez et al. 2019b), or 25.7% in *Clostridium perfringes* (Charlebois, Jacques and Archambault 2016) biofilms highlighting important differences in cell metabolism and the techniques used.

**Figure 4.**
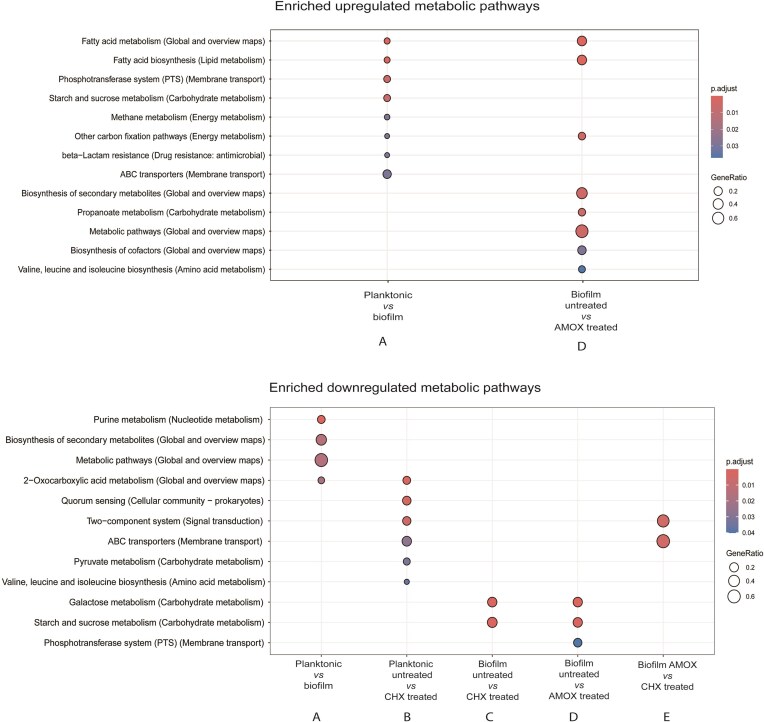
Up- and downregulated KEGG pathways enrichment results of the analysed conditions. The gene ratio represents the proportion of genes in a given pathway relative to the total number of genes in that pathway (*P*-value < 0.05). The size of the dots represents the count of genes coloured based on the adjusted *P-*value. Abbreviations: CHX, chlorhexidine, AMOX, amoxicillin.

**Figure 5.**
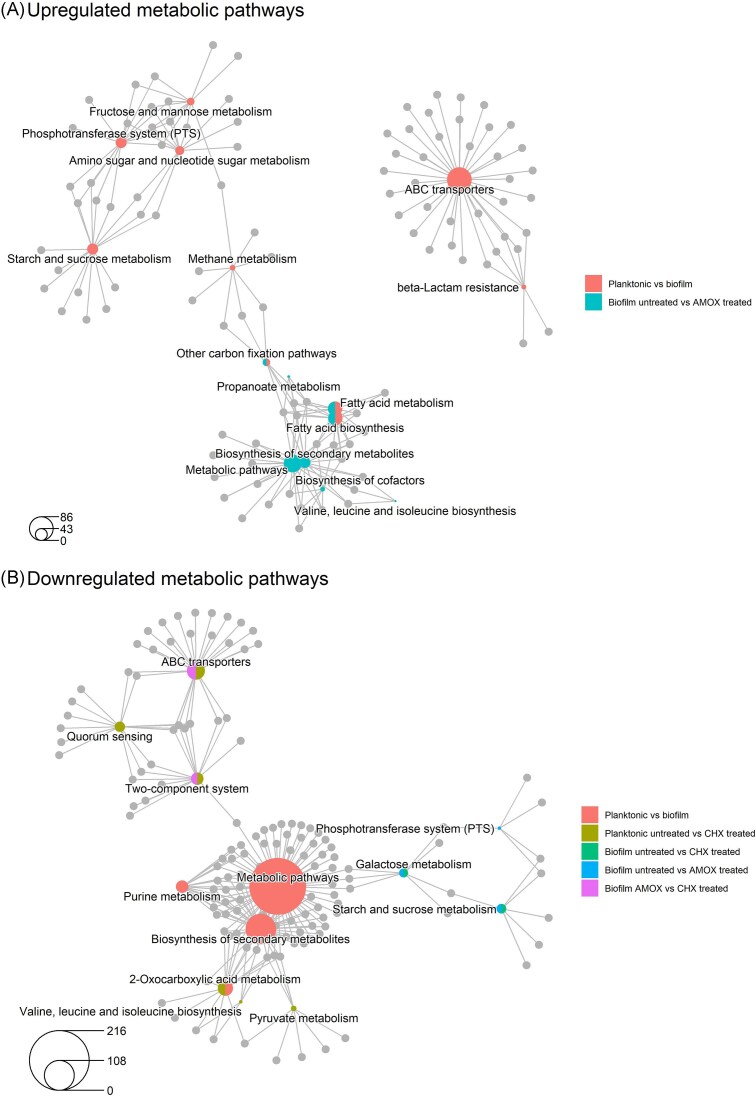
Cnet plots illustrating the enriched KEGG metabolic pathways and gene networks made with ClusterProfiler. The size of the circles in the legend represents the number of associated genes. **(A)** Upregulated metabolic pathways networ. (B) Downregulated metabolic pathways network.

To get an overview of the physiological processes affected in the *S. mutans* biofilms compared to planktonic cells, the expression patterns were examined by KEGG pathway enrichment analysis revealing several significantly up- and downregulated pathways (Fig. 4A, Table S4, Fig. 5). Fatty acid biosynthesis, phosphotransferase systems, starch and glucose metabolism, methane metabolism, β-lactam resistance, and ABC transporters were upregulated in the control biofilm compared to the planktonic cells (Figs. 4A and 5). On the other hand, purine metabolism and biosynthesis of secondary metabolites, metabolic pathways, and 2-oxocarboxylic acid metabolism within the general global and overview maps category were downregulated (Figs. 4 A and 5). The global biofilm gene expression pattern, representing the average across the heterogeneous biofilm cell population, showed a significant number of differentially regulated genes compared to the planktonic cells, indicating that biofilms exhibit distinct metabolic and physiological adaptations.

Figure 5 provides a visual summary of the up- and downregulated metabolic pathways across the experimental conditions, highlighting gene overlaps and multifunctional genes.

### The transcriptomic response to chlorhexidine treatment is dependent on the lifestyle of *S. mutans*

Chlorhexidine is the gold-standard oral antiseptic widely used in dental practice and as an over-the-counter mouthwash. Swallowing, expectoration, insufficient contact time, or concentration decline over time can lead to subinhibitory concentrations of chlorhexidine at target sites known to promote biofilm formation (Ebrahimi et al. 2014) and alter the metabolism and microbial composition of the oral microbiota (Chatzigiannidou et al. 2020).

Planktonic and biofilm-associated cells were treated with subinhibitory concentrations of chlorhexidine to evaluate its impact on the *S. mutans* UA159 transcriptome. Planktonic chlorhexidine-treated *S. mutans* cultures showed a total of 499 DEGs (225 upregulated, 274 downregulated genes, 24.4% of genes in total). In a similar study, Muehler et al. (2022) reported that treatment of *S. mutans* ATCC 25175 with 125-µg/mL chlorhexidine for 5 min resulted in 404 upregulated and 271 downregulated genes. Chlorhexidine-treated biofilms showed 100 DEGs (25 upregulated and 75 downregulated, 4.9%) (Fig. 2B) compared to the nontreated strain. Thus, as expected, there was a greater degree of differential regulation in chlorhexidine-exposed planktonic cells compared to chlorhexidine-exposed biofilms. This is illustrated in the heatmaps of the DEG distribution (Fig. 3A). These results are consistent with observations across different species showing that biofilm-associated cells are less metabolically active than their planktonic counterparts (Wan et al. 2018, Sadiq et al. 2020, Wang et al. 2023). Moreover, the 24-h mature biofilms tested in this study were likely in a state of nutrient limitation and therefore at a slow growth rate leading to a reduced metabolism.

Only 35 genes were found to be common between the planktonic and biofilm chlorhexidine-treated cells (Fig. 3B, Table S5). Those genes included ABC transporters, membrane proteins, the gene *comX1*, which is a key regulator of the natural competence system in streptococci (Aspiras et al. 2004, Kaspar et al. 2015), and mostly hypothetical genes with unknown functions.

#### Transcriptomic response in planktonic chlorhexidine-treated cultures

For the planktonic-chlorhexidine treated cultures, the global KEGG analysis revealed no enriched upregulated pathways, despite 225 genes being upregulated (Fig. 4B and C; Fig. 5, Table S4). On the other hand, quorum-sensing systems, two-component systems, and ABC-transporters, along with pyruvate metabolism, Val/Leu/Ile biosynthesis, and 2-oxocarboxylic acid metabolism, were downregulated according to the KEGG pathway analysis. Specifically, the regulatory genes included *ciaR-ciaH*, encoding the highly conserved streptococcal CiaRH regulatory system, which has been shown to be involved in natural competence, biofilm formation, bacteriocin production, and cell wall biosynthesis and autolysis (He et al. 2021). The *htrA* gene, encoding a CiaRH-regulated protease which takes part in oxidative stress tolerance, was also downregulated (Sebert et al. 2002, Ibrahim et al. 2004, He et al. 2021). Likewise, the gene *comE* is part of the ComCDE system, one of the natural competence pathways in *S. mutans* also involved in bacteriocin production (van der Ploeg 2005). Although the direct connection between these systems and chlorhexidine is unknown, one could speculate that membrane targeting agents such as chlorhexidine shift *S. mutans* metabolism to survival mechanisms not related to the CiaRH and ComCDE functions. The upregulated genes with highest fold-changes (|log_2_FC| > 5 and P_adj_ < 0.05, Table 2) included genes encoding hypothetical proteins and ABC transporters, such as the yet unstudied operon SMU_1550–SMU_1554, which represent prime candidates for further studies of factors affecting chlorhexidine sensitivity. Interestingly, this operon was also among the most highly upregulated operons upon exposure to a subinhibitory concentration of chlorhexidine in the study by Muehler et al. (2022) (locus tags D820_RS02750–D820_RS02735 in strain ATCC 25175, which was used in that study). Muehler et al. (2022) also reported widespread upregulation of stress response pathways and transport systems in planktonic *S. mutans* following exposure to a subinhibitory concentration of chlorhexidine, which to some extent aligns with genes shown to be upregulated in our study.

**Table 2.** Differentially expressed genes (|log_2_FC| > 5 and P_adj_ < 0.05).

Locus tag	log2FC	Description
*Planktonic vs planktonic chlorhexidine- treated*		
SMU_1093	−8.01	Putative ABC transporter, permease protein
SMU_1553c	−7.42	Hypothetical protein
SMU_1551c	−7.17	Putative ABC transporter, ATP-binding protein
SMU_1554c	−7.05	Hypothetical protein
SMU_1552c	−7.03	Hypothetical protein
SMU_1094	−7.01	Putative ABC transporter, ATP-binding protein
SMU_1131c	−6.66	Hypothetical protein
SMU_431	−6.48	Putative ABC transporter, ATP-binding protein
SMU_40	−5.78	Conserved hypothetical protein
SMU_41	−5.66	Hypothetical protein
SMU_1550c	−5.52	Conserved hypothetical protein; possible integral membrane protein
SMU_239c	−5.50	Hypothetical protein
SMU_739c	−5.50	Hypothetical protein
No upregulated genes with log_2_FC > 5		
*Biofilm vs. biofilm chlorhexidine-treated*		
SMU_1425	−5.07	*clpB*, putative Clp proteinase, ATP-binding subunitClpB
No upregulated genes with log_2_FC > 5		
*Biofilm vs biofilm amoxicillin treated*		
SMU_1425	−5.21	*clpB*, putative Clp proteinase, ATP-binding subunitClpB
No upregulated genes with log_2_FC > 5		

### The biofilm transcriptomic response is not specific to chlorhexidine

Chlorhexidine-treated biofilms showed no upregulated pathways compared to the untreated biofilms, with only carbohydrate metabolism (galactose, starch, and sucrose) being downregulated (Figs. 4C and 5). In a similar study testing the effect of inhibitory concentrations of curcumin and chlorhexidine on *S. mutans* biofilms, carbohydrate metabolism, quorum sensing, and two-component transduction systems were found downregulated (Li et al. 2018).

We were also interested in understanding whether the transcriptional remodelling in biofilms was specific to chlorhexidine. Amoxicillin is a relevant antibiotic in oral infection treatment. It is given as prophylaxis before oral intervention or for tooth infection treatment administered orally with a systemic effect that is expected to kill sessile bacteria prior to attachment and biofilm formation in the oral cavity. Amoxicillin was not effective in disrupting *S. mutans* UA159 biofilms with the used concentrations (up to 100 000-fold MIC), as observed in the BOAT assays (Fig. 1A and C). However, amoxicillin-treated biofilms still showed 149 DEGs (65 upregulated, 84 downregulated genes; 7.3% of genes in total) (Fig. 2C), compared to 100 DEGs for the chlorhexidine treatment. When directly comparing the transcriptomes of chlorhexidine-treated with the amoxicillin-treated biofilms, only 31 DEGs (3 upregulated, 28 downregulated; 1.5% of the total genes) were detected between the conditions (Fig. 2C), suggesting that the majority of the transcriptional responses to the individual agents in biofilms were not significantly different between the chlorhexidine and amoxicillin treatments, and rather represent general responses in the biofilm setting.

Interestingly, 68 commonly regulated genes were found between chlorhexidine- and amoxicillin-treated biofilms. The most downregulated ones were the same after chlorhexidine and amoxicillin treatments and included the phosphotransferase system-related genes *ptcA, ptsG, and mtlA1*, the *pdh* operon (pyruvate dehydrogenase) important during glucose starvation (Busuioc et al. 2010) and *naoX (noX)* that encodes the main enzyme in oxygen metabolism in *S. mutans* (Yamamoto et al. 2000, Derr et al. 2012) (Fig. 3B, Table S3, Table S5). Notably, the *clpB* gene (SMU_1425) exhibited the lowest log2FC value of −5.07 and −5.21 under both conditions (Table S3, Table 2). ClpB is a molecular chaperone part of the Clp ATPase family involved in homeostasis and stress tolerance (Lemos and Burne 2002, Frees et al. 2007) which might be reduced in biofilms compared to planktonic cells due to physiological adaptations (Stewart and Franklin 2008).

Furthermore, amoxicillin-treated biofilms showed downregulation of carbohydrate metabolism (galactose, starch, and sucrose) by the KEGG pathway analysis (Figs. 4D and 5), similar to what was observed in chlorhexidine-treated biofilms (Figs. 4 and 5). However, on the amoxicillin-treated biofilms, upregulated pathways that were not significantly different in the chlorhexidine treatment were detected (Fig. 4D, Table S4, Fig. 5). These included fatty acid metabolism and biosynthesis, carbon fixation, and branched-amino acids metabolism. Additionally, biosynthesis of secondary metabolites, propanoate metabolism, metabolic factors, and biosynthesis of cofactors were detected within the Global and overview maps category.

Overall, these observations suggest that there may be a common nonspecific biofilm response to chlorhexidine and amoxicillin.

### ABC transporters were differentially regulated across experimental conditions in *S. mutans*

ABC transporters play important roles in the active transport of molecules across the membrane for maintenance of cellular nutrient supply and integrity (Davidson et al. 2008). A large number of different ABC transporters were significantly regulated across the compared conditions tested here (Tables S6 and S7). Upon subinhibitory chlorhexidine exposure, 20% of the differentially regulated genes in broth cultures (10 out of 499) and 50% of those genes in biofilms (50 out of 100) were annotated as ABC transporter proteins. Considering that ABC transporter proteins in the annotated *S. mutans* UA159 reference genome are only 6.9% of the genes (141 out of 2043), ABC transporter genes were overrepresented among the regulated genes.

Among those whose functions are known, the *oppADF* genes, part of the *opp* operon responsible for oligopeptide uptake (Nepomuceno et al. 2007) and *msmFGK* and *malFX* genes involved in disaccharide uptake (Webb et al. 2008) were upregulated in *S. mutans* UA159 biofilm compared to its planktonic form and downregulated in chlorhexidine (*malX*) and amoxicillin-treated biofilms (*malX, malF*), whereas the *opu* genes *opuBa, opuBc*, and *opuCd* that regulate osmotic stress (Abranches et al. 2006) and the *mutF*, part of the MutEFG transporter that has been linked with nisin resistance in *S. mutans* (Le, Kawada-Matsuo and Komatsuzawa, 2022 ), were downregulated in the nontreated biofilms and after CHX-treated planktonic *S. mutans* UA159. Numerous transcriptomic studies report differential expression of ABC transporters in different bacterial species (Allan et al. 2014; Guo et al. 2022; Rahman et al. 2022; Rice et al. 2017; Zhu et al. 2008), highlighting their ubiquity and multifaceted nature. Up and downregulation of ABC transporters might be a response to counteract the stress induced by chlorhexidine and amoxicillin. In this sense, ABC transporters are known to be involved in the resistance and transport of antimicrobials (Abbood et al. 2023) and can function as antibiotic efflux pumps (Costa et al. 2013, Nagayama et al. 2014). Other functions of ABC transporters such as transport of diverse molecules (Biswas and Biswas 2011, Kim et al. 2012, Lemos et al. 2019) or nutrient uptake (McLaughlin and Ferretti 1996, Kilic et al. 2007, Webb et al. 2008) might as well be relevant in the response of *S. mutans* to chlorhexidine and amoxicillin.

## Conclusions

The results presented here provide a comprehensive overview of the transcriptomic response of *S. mutans* after exposure to subinhibitory concentrations of chlorhexidine and amoxicillin. Notably, these low concentrations of chlorhexidine and amoxicillin exerted a significant transcriptomic impact on planktonic and biofilm cultures of the oral commensal *S. mutans*. The observed changes in gene expression are likely to lead to phenotypic alterations, which could have implications for how this bacterium colonizes and responds to antibacterial treatments.

This compendium thus serves as a resource for further gene-targeted analysis to elucidate the roles of *S. mutans* genes that are differentially regulated under these settings. Combined treatment with chlorhexidine and amoxicillin could also be explored in future studies to assess potential additive or synergistic transcriptomic effects in oral biofilms, which may have important clinical implications.

## Supplementary Material

fnaf100_Supplemental_Files
