# Supplementary material for: Transcriptomic response in planktonic and biofilm-associated cells of Streptococcus mutans treated with sublethal concentrations of chlorhexidine
Source: FEMS Microbiol Lett. 2025 Sep 23;372:fnaf100. doi: 10.1093/femsle/fnaf100 (PMC12501421; doi:10.1093/femsle/fnaf100)
Supplement: fnaf100_Supplemental_Files [file fnaf100_supplemental_files.zip › Supplementary.docx]

**Supplementary figures**

**
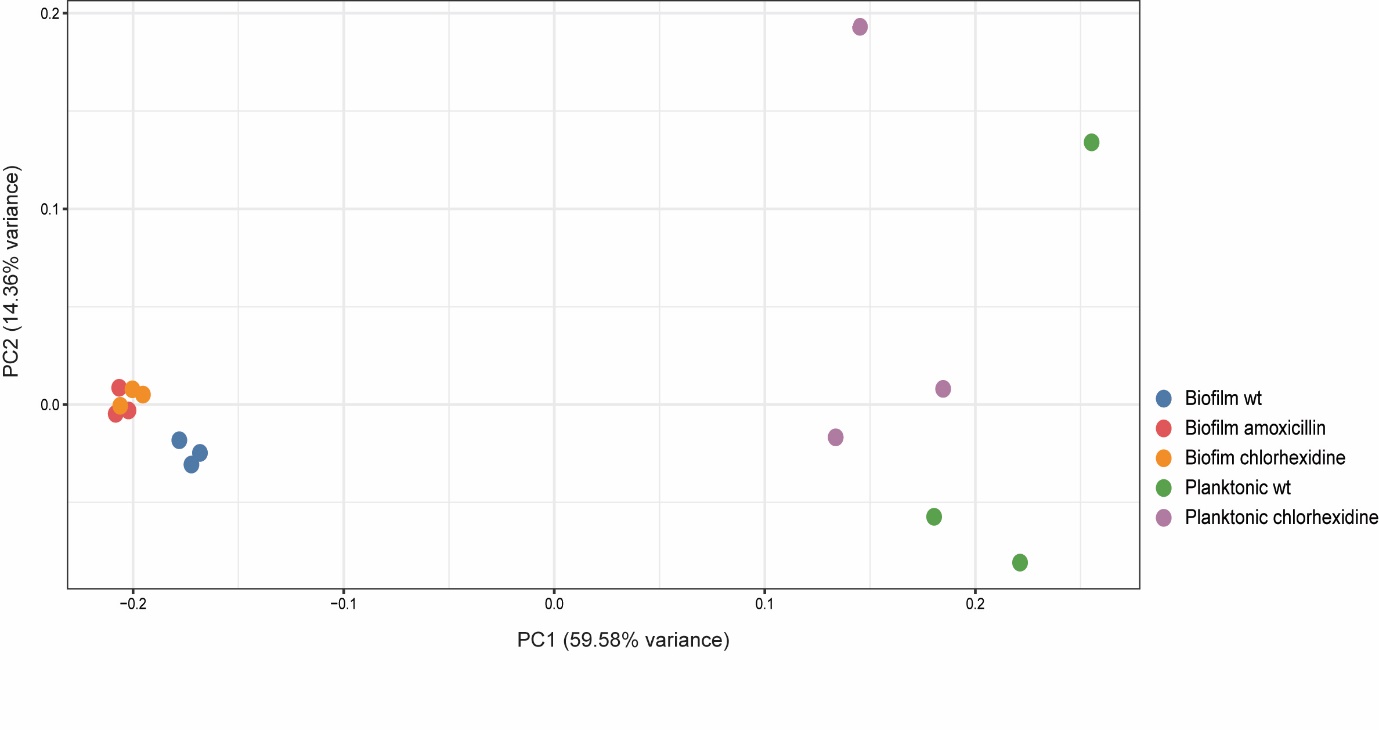
**

Figure S1. Principal component analysis (PCoA) based on Bray-Curtis dissimilarity. The plot illustrates the distances between the analysed conditions in all biological replicates. The number following B or P indicates the biological replicate. Abbreviations: wt, wild-type *S. mutans* UA159.


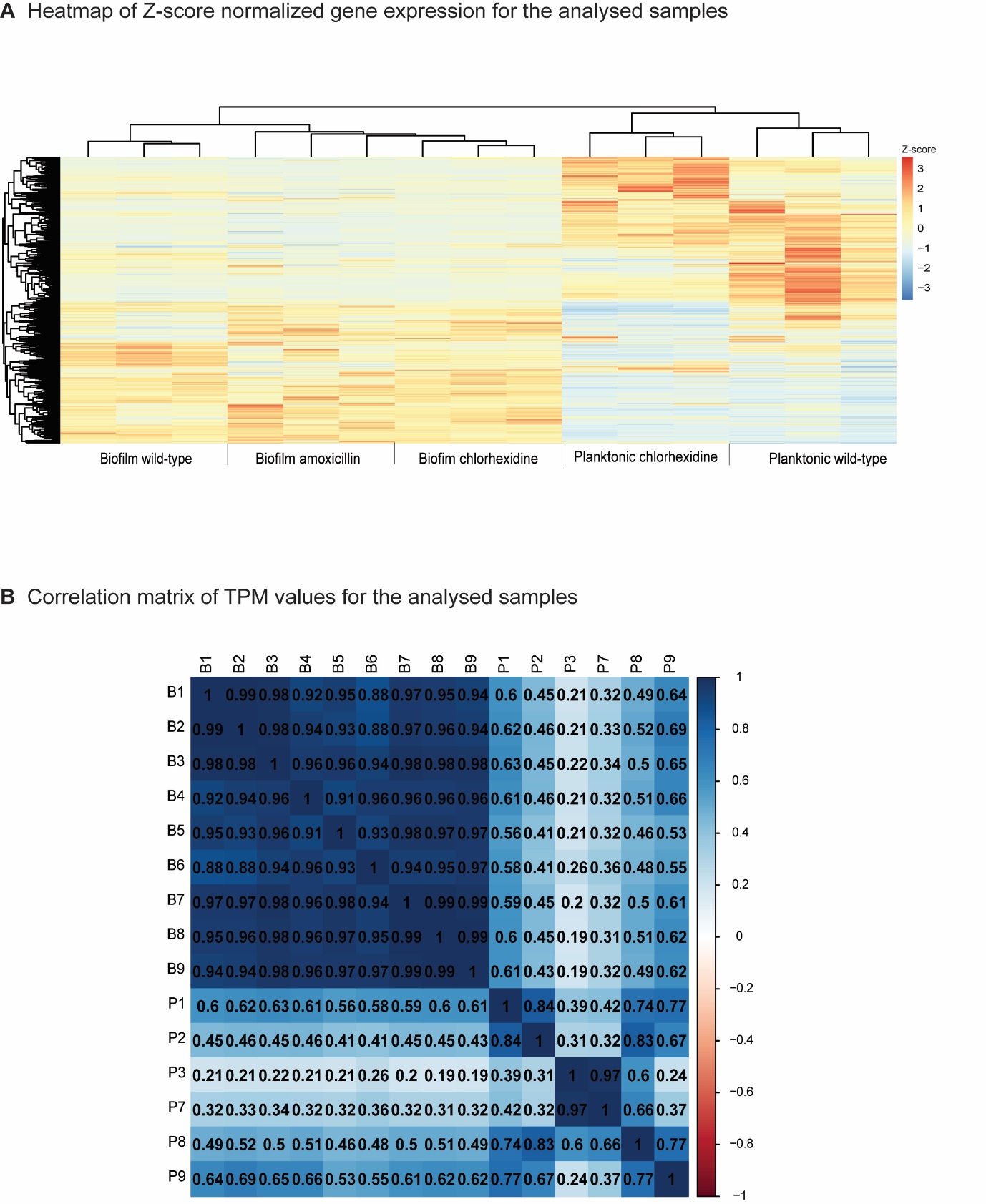


Fig S2. Quality assessment of the analysed samples based on TPM values. (A). Heatmap showing clustering of all genes and analysed samples. (B) Correlation plot showing the correlation coefficients between the analysed samples. Biofilm wild-type (B1-B3), biofilm amoxicillin (B4-B6), biofilm chlorhexidine (B7-B9), planktonic wild-type (P1-P3), planktonic chlorhexidine (P7-P9).

**Table S6**. Summary of significantly regulated ABC transporter and PTS system- related genes

| **Compared conditions** | **ABC transporters** |
| --- | --- |
| planktonic vs. biofilm | 38 up  25 down |
| planktonic vs. CHX^a^ planktonic | 22 up  28 down |
| biofilm vs. CHX biofilm | 1 up  9 down |
| biofilm vs. AMOX^b^ biofilm | 6 up  8 up |
| AMOX biofilm vs. CHX biofilm | 26 up  9 down |

^a^CHX: chlorhexidine, ^b^AMOX: amoxicillin
